# UVA Photoirradiation of Nitro-Polycyclic Aromatic Hydrocarbons—Induction of Reactive Oxygen Species and Formation of Lipid Peroxides ^†^

**DOI:** 10.3390/ijerph10031062

**Published:** 2013-03-14

**Authors:** Qingsu Xia, Jun J. Yin, Yuewei Zhao, Yuh-Sen Wu, Yu-Qui Wang, Liang Ma, Shoujun Chen, Xin Sun, Peter P. Fu, Hongtao Yu

**Affiliations:** 1 National Center for Toxicological Research, U.S. Food and Drug Administration, Jefferson, AR 72079, USA; E-Mails: qingsu.xia@fda.hhs.gov (Q.X.); ywzhao98@gmail.com (Y.Z.); yqqwang@yahoo.com (Y.-Q.W.); liang.ma@fda.hhs.gov (L.M.); schen@syntapharma.com (S.C.); 2 Center for Food Safety and Applied Nutrition, U.S. Food and Drug Administration, College Park, MD 20740, USA; E-Mail: Junjie.yin@fda.hhs.gov; 3 Hung Kuang University, Sha-Lu, Taichung, 443, Taiwan; E-Mail: ycwu@sunrise.hkc.edu.tw; 4 National Institute of Occupational Health and Poisoning Control, Chinese Center for Disease Control and Prevention, Beijing 100050, China; E-Mail: Xinsun10@sina.com; 5 Department of Chemistry and Biochemistry, Jackson State University, Jackson, MS 39217, USA

**Keywords:** nitro-polycyclic aromatic hydrocarbons, photoiradiation, UVA light, reactive oxygen species, lipid peroxidation

## Abstract

Nitro-polycyclic aromatic hydrocarbons (nitro-PAHs) are a class of genotoxic environmental contaminants. We have long been interested in determining the mechanisms by which nitro-PAHs induce genotoxicity. Although the metabolic activation of nitro-PAHs leading to toxicological activities has been well studied, the photo-induced activation of nitro-PAHs has seldom been reported. In this paper, we report photo-induced lipid peroxidation by 19 nitro-PAHs. The results indicated that all but two of the nitro-PAHs can induce lipid peroxidation. Mechanistic studies suggest that lipid peroxidation by nitro-PAHs is mediated by free radicals generated in the reaction. There was no structural correlation between the nitro-PAHs and their ability to induce lipid peroxidation upon UVA irradiation, or between the HOMO-LUMO gap and the ability to cause lipid peroxidation. Most of the nitro-PAHs are less potent in terms of causing lipid peroxidation than their parent PAHs. The lack of correlation is attributed to the complex photophysics and photochemistry of the nitro-PAHs and the yield of reactive oxygen species (ROS) and other factors.

## 1. Introduction

Since 1978, nitro-polycyclic aromatic hydrocarbons (nitro-PAHs) have been identified as widespread genotoxic environmental contaminants from different environmental sources, including diesel emissions, combustion emissions from kerosene heaters, gas fuel, and liquefied petroleum, airborne particulates, coal fly ash, and food [1,2,3,4,5,6]. Nitro-PAHs require metabolic activation in order to exert their mutagenic and carcinogenic activities [7,8,9,10,11,12]. Because of their widespread presence in the environment and genotoxic activities, many of these compounds may pose a health risk to humans. The environmental occurrence and toxicological activities, including metabolism, mutagenicity, and carcinogenicity, of a large number of nitro-PAHs have been extensively studied [5,7,11].

**Figure 1:**
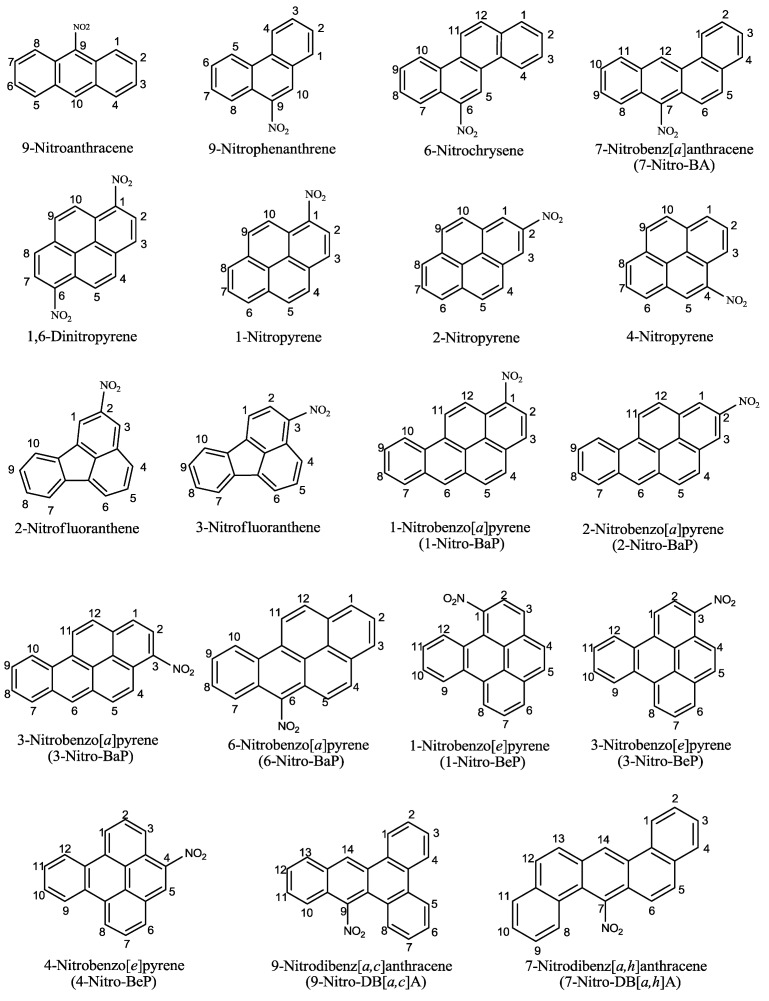
Structures, names, numberings, and abbreviations of nitro-PAHs used in this study.

We have long been interested in studying nitro-PAH induced mutagenesis and carcinogenesis on the basis of structure-activity relationship [12,13,14,15,16,17,18]. To date, the study on nitro-PAH induced phototoxicity on the basis of structure-activity relationship has been limited [14,19,20]. There has no systematic report on the study of induction of lipid peroxidation by photoirradiation of nitro-PAHs on a basis of structure-activity relationships. This is because, in most cases, the structurally related nitro-PAHs are not available for study. In this paper we report: (i) novel synthesis of 1-nitrobenzo[*a*]pyrene (1-nitro-BaP), 3-nitro-BaP, 1-nitrobenzo[*e*]pyrene (1-nitro-BeP), and 3-nitro-BeP; (ii) UVA photoirradiation of nineteen representative nitro-PAHs and ten parent PAHs (Figure 1) in the presence of methyl linoleate, which resulted in lipid peroxide (methyl linoleate hydroperoxides) formation; and (iii) use of enzyme inhibition and electron spin resonance (ESR) spin trapping technique to determine the formation of free radicals, including reactive oxygen species. ESR spin trapping studies indicated that singlet oxygen, superoxide anion radicals, and nitro-PAH-derived free radicals were generated from UVA photoirradiation of nitro-PAHs in a light dose dependent manner. The formation of nitro-PAH-induced lipid peroxidation by UVA irradiation is also discussed on the basis of structure-activity relationships.

## 2. Materials and Methods

### 2.1. Materials and Instrumentation

Anthracene, phenanthrene, benz[*a*]anthracene (BA), chrysene, pyrene, fluoranthene, benzo[*a*]pyrene (BaP), benzo[*e*]pyrene (BeP), dibenz[*a*,*h*]anthracene (DB[*a*,*h*]A), dibenz[*a,c*]-anthracene (DB[*a*,*c*]A), 1-nitronaphthalene, 9-nitroanthracene, sodium borohydride, trifluoroacetic acid, p-toluenesulfonic acid (TsOH), sodium nitrate, anhydrous hydrazine, 2,3-dichloro-5,6-cyano-1,6-benzoquinone (DDQ), sodium azide (NaN_3_), dithiothreitol (DTT), superoxide dismutase (SOD), methyl linoleate, 5,5-dimethyloxide pyrroline (DMPO), and 2,2,6,6-tetramethylpiperidine (TEMP) were purchased from Sigma-Aldrich (St. Louis, MO, USA). The nitrone spin trap, 5-*tert*-butoxycarbonyl-5-methyl-1-pyrroline-N-oxide (BMPO) was purchased from Applied Bioanalytical Labs (Sarasota, FL, USA). 2-Nitro-BaP was synthesized as previously described [21]. 1-Nitro-BaP, 3-nitro-BaP, 6-nitro-BaP, 1-nitro-BeP, and 3-nitro-BeP were prepared through new synthesis methods described below. All the other nitro-PAHs used in this study were prepared as described previously [18]. All the nitro-PAHs and PAHs were analyzed by HPLC and found to be >99% pure. Other chemicals and solvents were purchased from either Sigma-Aldrich (St. Louis, MO, USA) or Fisher Scientific (Houston, TX, USA).

A Waters HPLC system consisting of a Model 600 controller, a Model 996 photodiode array detector, and pump was used to determine the amount of methyl linoleate hydroperoxide formed. Conventional ESR spectra were obtained with a Varian E-109 X-band spectrometer and interfaced to a personal computer for data acquisition and manipulation. ESR signals were recorded with 15 mW incident microwave and 100 kHz field modulation of 1.25 G (for TEMP) and 1G (for DMPO-OOH). The scan ranges were 80 G (for TEMP) and 100 G (for DMPO-OOH), respectively. All measurements were performed at room temperature. For organic synthesis, all mass spectra analyses were performed on a JEOL JMS‑DX300 mass spectrometer with a solid probe by electron impact at 70 eV. UV-Vis absorption spectra were obtained using a Shimadzu UV-260 spectrophotometer, and samples were dissolved in methanol. Proton NMR spectra were measured in acetone‑d_6 _with a Bruker WM-500 spectrometer.

### 2.2. Synthesis of 1-, 3-, and 6-Nitrobenzo[a]pyrene (Scheme 1)

Nitration of 10-keto-7,8,9,10-tetrahydrobenzo[*a*]pyrene [22] (950 mg, 3.52 mmol) in glacial acetic acid (150 mL) with equimolar sodium nitrate was carried out in trifluoroacetic acid (TFA, 25 mL) at ice-water temperature for 30 min, followed by silica gel column chromatography using 20% ethyl acetate in hexane to furnish pure 1-nitro-10-keto-7,8,9,10-tetrahydro-BaP (**1**), 3-nitro-10-keto-7,8,9,10-tetrahydro-BaP (**2**), and 6-nitro-10-keto-7,8,9,10-tetrahydro-BaP (**3**) in that order.

*1-Nitro-10-keto-7,8,9,10-tetrahydro-BaP* (**1**): 26% yield, m.p. 237–238°; mass spectrum, *m/z* 315 (M^+^); UV-Vis spectrum, 389 (e = 1,300), 335 (4,000) and 320 nm (5,400);
*3-Nitro-10-keto-7,8,9,10‑tetrahydro-BaP* (**2**): 23% yield, m.p. 250–251.5°; mass spectrum, *m/z* 315 (M^+^); UV-Vis spectrum, 384 (11,000), 290 (1,500) and 280 (1,500);
*6-Nitro-10-keto-7,8,9,10-tetrahydro-BaP* (**3**): 45% yield, m.p. 212–213°; mass spectrum, *m/z* 315 (M^+^); UV-Vis spectrum, 396 (1,000), 377 (14,000), 366 (1,400), 209 (1,900), 233 (3,100) and 203 (2,400).

1-Nitro-10-keto-7,8,9,10-tetrahydro-BaP (**1**, 248 mg) in tetrahydrofuran (40 mL) was reduced with sodium borohydride (150 mg) in methanol (45 mL) at ambient temperature for 1 hr. The reaction product was poured into ice water and neutralized with hydrochloric acid, affording the alcohol 1-nitro-10-hydroxy-7,8,9,10-tetrahydro-BaP (**4**) in 92% yield; mp 157–158°; mass spectrum, *m/z* 317 (M^+^); UV-Vis spectrum, 398 (44,500), 290 (15,300) and 239 (57,000). Dehydration of 1-nitro-10-hydroxy-7,8,9,10-tetrahydro-BaP (**4**, 231 mg) in refluxing benzene catalyzed by *p*-toluenesulfonic acid gave 1-nitro-7,8-dihydro-BaP (**5**) in 96% yield; mp 179–180°; mass spectrum, *m/z* 299 (M^+^). Dehydrogenation of 1-nitro-7,8-dihydro-BaP (**5**, 100 mg) with an equal molar DDQ in refluxing benzene for 8 h gave 1-nitro-BaP in nearly quantitative yield; m.p. 250–251° (lit. [23] m.p. 250–250.5°); mass spectrum, *m/z* 297 (M^+^); NMR (acetone-d_6_) 7.94 (dd, 1, H_8_), 8.02 (dd, 1, H_9_), 8.19 (d, 1, H_4_), 8.36 (d, 1, H_3_), 8.39 (d, 1, H_5_), 8.49 (d, 1, H_7_), 8.77 (d, 1, H_2_), 8.94 (s, 1, H_6_), 9.12 (d, 1, H_12_), 9.32 (d, 1, H_10_) and 9.57 ppm (d, 1, H_11_); J_2,3_ = 8.3 Hz, J_4,5_ = 9.2 Hz, J_7,8_ = 8.1 Hz, J_8,9_ = 6.9 Hz, J_9,10_ = 8.6 Hz, and J_11,12_ = 9.6 Hz.

**Scheme 1:**
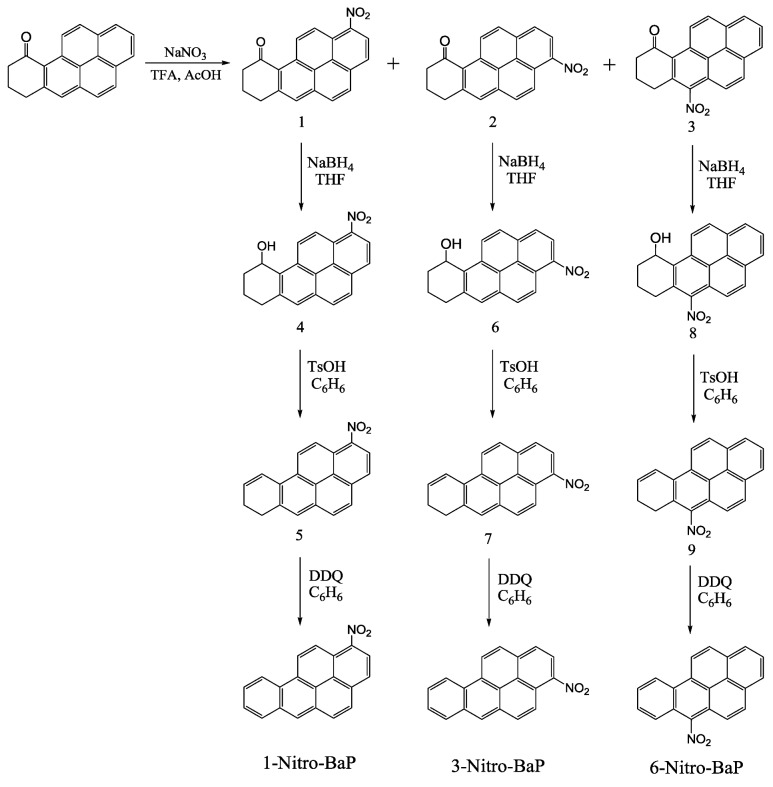
Synthesis of 1-, 3-, and 6-nitrobenzo[*a*]pyrene.

Similarly, 3-nitro-10-keto-7,8,9,10‑tetrahydro-BaP (**2**) was reduced to the corresponding alcohol 3-nitro-10-hydroxy-7,8,9,10-tetrahydro-BaP (**6**) (98% yield, mp 246–247°), which upon acid-catalyzed dehydration afforded 3-nitro-7,8-dihydro-BaP (**7**) in 96% yield (mp 179–180°). Dehydrogenation of 3-nitro-7,8-dihydro-BaP (100 mg) with DDQ gave 3-nitro-BaP in a 99% yield; mp 211‑212° (lit. [23] mp 211–212°); mass spectrum, m/z 297 (M^+^); NMR (acetone-d_6_) 7.94 (dd, 1, H_8_), 8.01 (dd, 1, H_9_), 8.47 (d, 1, H_5_), 8.49 (d, 1, H_7_), 8.50 (d, 1, H_1_), 8.59 (d, 1, H_12_), 8.63 (d, 1, H_2_), 8.64 (d, 1, H_4_), 8.94 (s, 1, H_6_), 9.31 (d, 1, H_10_) and 9.47 ppm (d, 1, H_11_); J_1,2_ = 8.6 Hz, J_4,5_ = 9.5 Hz, J_7,8_ = 8.1 Hz, J_8,9_ = 6.8 Hz, J_9,10_ = 8.5 Hz, and J_11,12_ = 9.2 Hz. Starting with 6-nitro-10-keto-7,8,9,10-tetrahydro-BaP (**3**), 6-nitro-BaP was similarly obtained.

### 2.3. Synthesis of 1- and 3-Nitrobenzo[e]pyrene (Scheme 2)

**Scheme 2:**
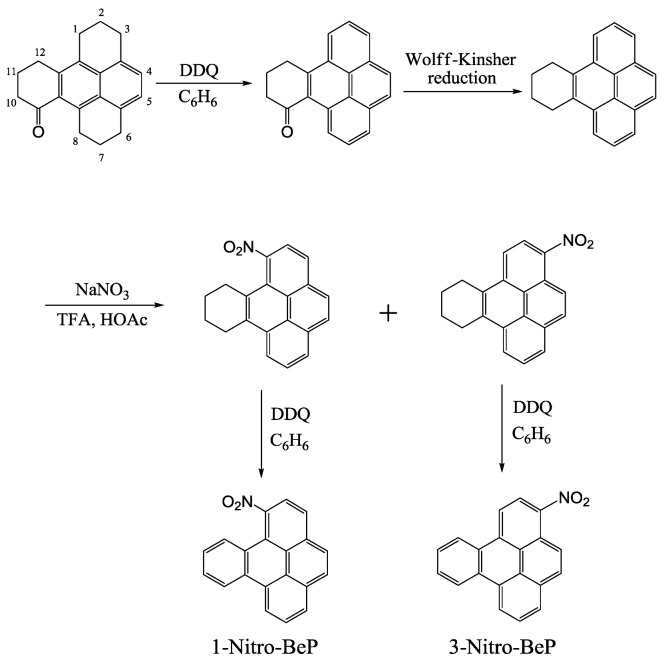
Synthesis of 1- and 3-nitrobenzo[*e*]pyrene.

9-Keto-1,2,3,6,7,8,9,10,11,12-decahydro-BeP was synthesized by succinylation of 1,2,3,6,7,8-hexahydropyrene, followed by Clemmenson reduction of the resulting keto-acid and acid-catalyzed ring cyclization. Reduction of 9-keto-1,2,3,6,7,8,9,10,11,12-decahydro-BeP with three molar equivalent of DDQ in benzene under reflux for 1 h formed 9-keto-9,10,11,12-tetrahydro-BeP (Scheme 2). Wolff-Kishner reduction of 9-keto-9,10,11,12-tetrahydro-BeP dissolved in *n*-butane with anhydrous hydrazine under reflux for 48 h afforded 9,10,11,12‑tetrahydro-BeP; mass spectrum, *m/z* 256 (M^+^); NMR (acetone-d_6_) 8.38 (d, 2, H_1,8_), 8.24 (d, 2, H_3,6_), 8.15 (d, 2, H_4,5_), 8.06 (d, 2, H_2,7_), 3.35 (m, 2, H_9,12_) and 2.11 ppm (m, 2, H_10,11_); J_1,2_ = J_2,3_ = J_6,7_ = J_7,8_ = 7.7 Hz.

Nitration of 9,10,11,12-tetrahydro-BeP (100 mg, 0.39 mmol) was carried out in glacial acetic acid (50 mL) at ice-water temperature under argon with NaNO_2_ (35 mg, 0.41 mmol) in trifluoroacetic acid (17 mL) for 6 h. Silica gel column chromatography using 33% benzene in hexane furnished 1-nitro-9,10,11,12-tetrahydro-BeP and 3-nitro-9,10,11,12-tetrahydro-BeP; *1-nitro-9,10,11,12-tetrahydro-BeP*—mass spectrum, *m/z* 301 (M^+^); NMR (acetone-d_6_) 8.60 (dd, 1, H_8_), 8.44 (dd, 1, H_6_), 8.38 (d, 1, H_3_), 8.35 (d, 1, H_4_), 8.28 (d, 1, H_2_), 8.27 (d, 1, H_5_), 8.21 (d, 1, H_7_), 3.51 (m, 2, H_9_), 3.07 (m, 2, H_12_), 2.15 (m, 2, H_10_) and 1.88 ppm (m, 2, H_11_); J_2,3_ = 8.1 Hz, J_4,5_ = 8.8 Hz, J_6,7_ = 7.7 Hz, and J_7,8_ = 7.7Hz. *3-nitro-9,10,11,12-tetrahydro-BeP*—mass spectrum, *m/z* 301 (M^+^); NMR (acetone-d_6_) 8.77 (d, 1, H_4_), 8.71 (d, 1, H_2_), 8.61 (dd, 1, H_8_), 8.51 (d, 1, H_1_), 8.47 (d, 1, H_5_), 8.44 (dd, 1, H_6_), 8.22 (d, 1, H_7_), 3.45 (d, 2, H_9_), 3.39 (dd, 2, H_12_), and 2.13 ppm (dd, 4, H_10,11_); J_1,2_ = 8.8 Hz, J_4,5_ = 9.4 Hz, J_6,7_ = 7.9 Hz, and J_7,8_ = 7.9 Hz.

Dehydrogenation of 1‑nitro‑9,10,11,12-tetrahydro‑BeP (100 mg) with DDQ gave 1-nitro-BeP in 96% yield; mass spectrum, *m/z* 297 (M^+^); NMR (acetone‑d_6_) 9.20 (dd, 1, H_8_), 9.10 (dd, 1, H_9_), 8.46 (d, 1, H_3_), 8.45 (dd, 1, H_6_), 8.36 (d, 1, H_5_), 8.33 (d, 1, H_2_), 8.28 (d, 1, H_4_), 8.25 (dd, 1, H_12_), 8.24 (dd, 1, H_7_), 7.90 (dd, 1, H_10_) and 7.74 ppm (dd, 1, H_11_); J_2,3_ = 8.3 Hz, J_4,5_ = 9.0 Hz, J_6,7_ = 7.7 Hz, J_7,8_ = 8.1 Hz, J_9,10_ = 8.3 Hz, J_10,11_ = 7.1 Hz, and J_11,12_ = 8.4 Hz. Dehydrogenation of 3-nitro-9,10,11,12-tetra-hydro-BeP (100 mg) with DDQ gave 3-nitro-BeP in 99% yield; mass spectrum, m/z 297 (M^+^); NMR (acetone‑d_6_) 9.26 (dd, 1, H_8_), 9.25 (d, 1, H_1_), 9.10 (dd, 1, H_12_), 9.09 (dd, 1, H_9_), 8.73 (d, 1, H_2_), 8.70 (d, 1, H_4_), 8.48 (d, 1, H_5_), 8.45 (dd, 1, H_6_), 8.26 (dd, 1, H_7_), 7.95 (dd, 1, H_11_) and 7.90 ppm (dd, 1, H_10_); J_1,2_ = 9.0 Hz, J_4,5_ = 9.5 Hz, J_6,7_ = 8.2 Hz, J_7,8_ = 7.3 Hz, J_9,10_ = 8.2 Hz, and J_11,12_ = 8.2 Hz.

### 2.4. Light Sources

The UVA light box was custom made using four UVA lamps (National Biologics, Twinsburg, OH, USA) [23]. Spectral irradiance of the light box was determined using an Optronics OL754 Spectroradiometer (Optronics Laboratories, Orlando, FL, USA), and the light dose was routinely measured using a Solar Light PMA-2110 UVA detector (Solar Light Inc., Philadelphia, PA, USA). The maximum emission of the UVA light box was determined to be between 340–355 nm. The light intensities at wavelengths below 320 nm (UVB light) and above 400 nm (visible light) are approximately two orders of magnitude lower than the maximum in the 340 – 355 nm spectral regions [23].

The UVA-irradiation doses used for study were from 7 - 35 J/cm^2^, approximately 23 to 115 min exposure at the dose rate of 5 mW/cm^2^. Ten J/cm^2^ of UVA, equals to about 2 h exposure at the noon time of sunny days during the summer around the world, based upon observations of UVA intensity of 2.1 mW/cm^2^ in Okayama, Japan in September [24], 3.6 mW/cm^2^ in Jackson, MS, USA in August [25], 5.4 mW/cm^2^ in Paris, France in July [26], 6.6 mW/cm^2^ in Coimbatore, India in July [27].

### 2.5. Peroxidation of Methyl Linoleate by UVA Photoirradiation of Nitro-PAHs

Experiments were conducted using a solution of 100 mM methyl linoleate and 1.0 mM nitro-PAH in methanol. Samples were placed in a UV-transparent cuvette and irradiated with 0, 7, or 21 J/cm^2^ of UVA light. After irradiation, the methyl linoleate hydroperoxides were separated by HPLC using a Prodigy 5 µm ODS column (4.6 × 250 mm, Phenomenex, Torrance, CA, USA) eluted isocratically with 10% water in methanol (v/v) at 1 mL/min. The levels of lipid peroxidation were determined by HPLC, and levels were quantified by monitoring the elution of HPLC peak areas at 235 nm [23,28] followed by conversion to the concentration based on the molar extinction coefficient (at 235 nm) reported before [29]. The 10 parent PAHs were also studied in parallel.

### 2.6. Peroxidation of Methyl Linoleate Initiated by Photoirradiation of Nitro-PAHs in the Presence of a Free Radical Scavenger or Enhancer

The experiments were carried out as described above, with the exception that parallel experiments were conducted with UVA irradiation at 0, 7, 21, or 35 J/cm^2^ of UVA light and in the presence NaN_3_ or SOD. The concentration of SOD was 200 U/mL and NaN_3_ was 20 mM. It has been established that the lifetime of singlet oxygen is longer in deuterated solvents, such as deuterated water or methanol, than in protic solvent [30]. The effect on the levels of lipid peroxide formation induced by UVA photoirradiation of nitro-PAHs in CH_3_OH and CH_3_OD was conducted similarly.

### 2.7. Detection of Reactive Oxygen Species and Free Radicals by Photoirradiation of Nitro-PAHs Using ESR with Spin Trapping

ESR with spin trapping was used to detect superoxide anion radicals (O_2_^−^·) formed by photoirradiation of nitro-PAHs at fixed time points. Each nitro-PAH at 0.9 mM in 90% CH_3_CN was mixed with 25 mM BMPO [31,32] and transferred to a 50 µL quartz capillary tube. The capillary tube was placed into the microwave cavity of a Bruker EMX ESR Spectrometer (Billerica, MA, USA). Nitro-PAH (4-nitropyrene or 4-nitro-BaP) was irradiated at wavelength 420 nm in the microwave cavity using light emitted from a 500 W Xe Arc lamp directed through a McPherson monochromator, model DM200 (Chelmsford, MA, USA). ESR spectra were collected at irradiation times of 5, 12, 20, 30, and 40 min. All ESR measurements were carried out at ambient temperature (27 °C) using the following settings for detection of the spin adduct between BMPO and O_2_^−^· (DMPO-OOH ): 20 mW microwave power, 100 G scan range and 1 G field modulation amplitude, and 100 kHz modulation frequency. The data were obtained with error of less than 10%. For detection of singlet oxygen, samples containing 0.9 mM nitro-PAH and 20 mM TEMP in 95% CH_3_CN were irradiated at 340 nm, and ESR spectra were recorded after 20, 30, and 40 min of irradiation, respectively. Control did not contain any nitro-PAH.

### 2.8. Quantum Chemical Calculation of LUMO/HOMO Energy Gap

Quantum chemical calculations were carried out using the Gaussian 09 program package [33]. The geometry optimization (with Tight and GDIIS being options of the optimizer) was conducted using DFT (B3LYP hybrid functional) method with the 6–31G (d) basis set. The solvent effects were considered using the polarized continuum model which is performed with self-consistent reaction field in Gaussian. Methanol was employed for simulating real experimental conditions. The energy gaps between the highest occupied molecular orbital (HOMO) and the lowest unoccupied molecular orbital (LUMO) of ten nitro-PAHs were calculated.

## 3. Results

### 3.1. Synthesis of Nitro-BaPs and Nitro-BePs

Toxicological studies, including phototoxicity, require pure substrates for experiments. To our understanding, the synthesis of 1-, 3-, 6-nitro-BaP, 1- and 3-nitro-BeP, as shown in Scheme 1 and Scheme 2, represents the best approach so far reported. Our synthetic method provides all these compounds with high yield and purity, and the isomers are easily isolated by column chromatography. Purity and structural assignments were achieved by the usual array of analytical techniques such as ^1^H-NMR spectroscopic, mass spectroscopy, melting points, and UV-Vis. Among these nitro-PAHs, 1-nitro-BaP, 3-nitro-BaP, and 3-nitro-BeP have a parallel orientation for their nitro groups, while 6-nitro-BaP and 1-nitro-BeP place their nitro groups in a perpendicular orientation to the aromatic ring. It has been reported that the nitro orientation is a major factor for the toxicological activities of nitro-PAHs [14,18]. Thus, these isomeric nitro-PAHs with the same molecular weight but different geometric structures with nitro functional group at different locations and orientations are ideal models for studying structure-activity relationships of nitro-PAHs.

### 3.2. UVA Photoirradiation of Nitro-PAHs and Their Parent PAHs in the Presence of Methyl Linoleate

A total of 19 structurally related nitro-PAHs were selected for the study of photoirradiation with UVA in the presence of a lipid, methyl linoleate. These nitro-PAHs are derived from the parent PAHs including anthracene, phenanthrene, pyrene, benz[*a*]anthracene (BA), chrysene, fluoranthene, BaP, BeP, dibenz[*a*,*h*]anthracene (DB[*a*,*h*]A), and dibenz[*a*,*c*]anthracene (DB[*a*,*c*]A) (Figure 1). These selected nitro-PAHs possess different sizes, ranging from three to five fused benzene-rings, and exhibit various carcinogenic potency. For comparison, ten parent PAHs are also included for this study. Each compound received two light doses of 7 and 21 J/cm^2^. Under these experimental conditions, among the 19 nitro-PAHs tested, there are 17 nitro-PAHs that induced lipid peroxidation; 1-nitro-BeP and 3-nitro-BeP are the only nitro-PAHs that did not induce lipid peroxidation (Table 1). Upon UVA irradiation, with the exception of phenanthrene, all parent PAHs also induced lipid peroxidation (Table 1).

**Figure 2:**
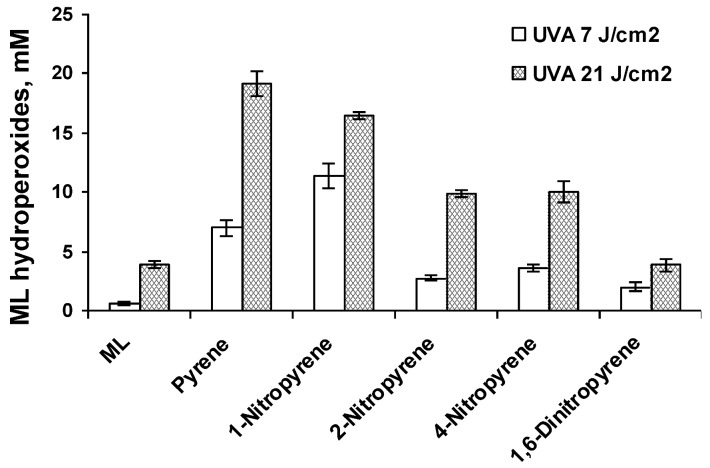
Lipid peroxidation induced by photoirradiation of pyrene, 1-nitropyrene, 2-nitropyrene, 4-nitropyrene, and 1,6-dinitropyrene in methanol in the presence of methyl linoleate (ML) with UVA light at a light dose of 7 and 21 J/cm^2^ respectively. The levels of peroxidation were measured by HPLC analysis monitoring the elution at 235 nm.

**Figure 3:**
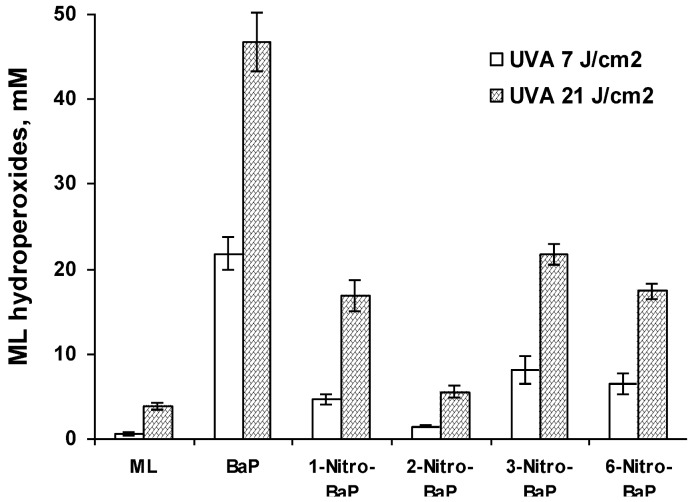
Lipid peroxidation induced by photoirradiation of BaP, 1-nitro-BaP, 2-nitro-BaP, 3-nitro-BaP, and 6-nitro-BaP in methanol in the presence of methyl linoleate (ML) with UVA light at a light dose of 7 and 21 J/cm^2^ respectively.

**Figure 4:**
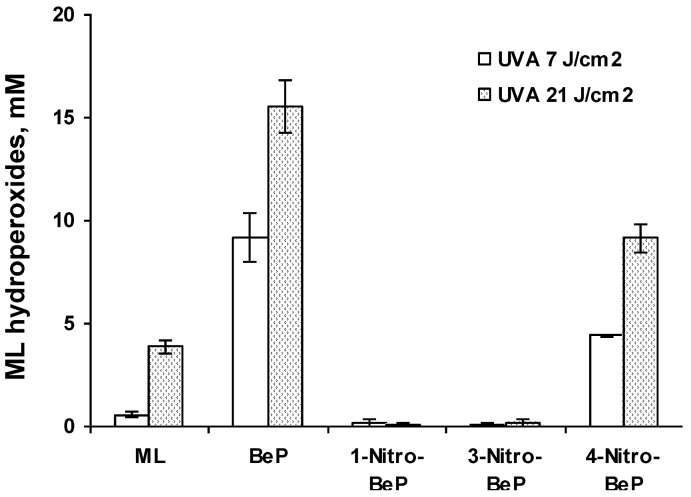
Lipid peroxidation induced by photoirradiation of BeP, 1-nitro-BeP, 3-nitro-BeP, and 4-nitro-BeP in methanol in the presence of methyl linoleate (ML) with UVA light at a light dose of 7 and 21 J/cm^2^ respectively.

As summarized in Table 1, the resulting lipid peroxidation exhibited a light-dose response. In most cases, the parent PAHs, including pyrene (Figure 2), BaP (Figure 3), and BeP (Figure 4), induced lipid peroxidation higher than their corresponding nitro-PAHs. The levels of lipid peroxidation induced by the nitro-PAH isomers were also drastically different. As shown in Figure 2, the order of lipid peroxidation induced by pyrene and nitropyrenes is: pyrene ≥ 1-nitropyrene > 2-nitropyrene ≈ 4-nitro-pyrene > 1,6-dinitropyrene. In the BeP series, both 1-nitro-BeP and 3-nitro-BeP did not induce lipid peroxidation, and 4-nitro-BeP induced lipid peroxidation at a level lower than that from BeP (Figure 4). As to the BaP and nitro-BaP series, the results shown in Figure 3 indicate the level of induction in an order of BaP > 1-nitro-BaP ≈ 3-nitro-BaP ≈ 6-nitro-BaP > 2-nitro-BaP.

**Table 1:** Generation of methyl linoleate hydroperoxide by UVA irradiation of nitro-PAHs and PAHs in the presence of methyl linoleate (ML).

Number of rings	Compound Name	Methyl linoleate hydroperoxide (peak area with a maximum at 235 nm) UVA 7 J/cm^2 ^UVA 21 J/cm^2^	
	Negative Control (ML only)	289 ± 34	479 ± 30
3 rings	**Anthracene**	1,690 ± 84	3,390 ± 125
	9-Nitroanthracene	1,557 ± 55	3,106 ± 50
	**Phenanthrene**	323 ± 52	603 ± 78
	9-Nitrophenanthrene	799 ± 54	1,412 ± 52
4-rings	**Pyrene**	1,044 ± 81	2,601 ± 129
	1-Nitropyrene	1,612 ± 158	2,256 ± 54
	2-Nitropyene	492 ± 16	1,400 ± 51
	4-Nitropyene	610 ± 19	1,438 ± 91
	**Chrysene**	1,082 ± 192	2,002 ± 175
	6-Nitrochrysene	1,192 ± 69	2,144 ± 215
	**Benz[*a*]anthracene**	2,785 ± 125	4,390 ± 273
	7-Nitrobenz[ *a*]anthracene	996 ± 39	2,096 ± 233
	**Fluoranthene**	1,413 ± 147	3,522 ± 442
	2-Nitrofluoranthene	1,581 ± 109	2,908 ± 249
	3-Nitrofluoranthene	924 ± 74	1,788 ± 187
5-rings	**Benzo[*a*]pyrene**	2,972 ± 257	6,153 ± 462
	1-Nitrobenzo[ *a*]pyrene	753 ± 88	2,319 ± 241
	2-Nitrobenzo[ *a*]pyrene	346 ± 8	858 ± 78
	3-Nitrobenzo[ *a*]pyrene	1,205 ± 198	2,937 ± 142
	6-Nitrobenzo[ *a*]pyrene	1,003 ± 143	2,387 ± 136
	**Benzo[*e*]pyrene**	1,341 ± 155	2,155 ± 169
	1-Nitrobenzo[ *e*]pyrene	171 ± 15	163 ± 2
	3-Nitrobenzo[ *e*]pyrene	169 ± 6	179 ± 13
	4-Nitrobenzo[ *e*]pyrene	724 ± 13	1,372 ± 85
	**Dibenz[*a*,*h*]anthracene**	1,446 ± 107	2,835 ± 257
	7-Nitrodibenz[ *a*,*h*]anthracene	904 ± 22	2,365 ± 54
	**Dibenz[*a*,*c*]anthracene**	2,091 ± 190	3,935 ± 315
	9-Nitrodibenz[ *a*,*c*]anthracene	1,025 ± 97	2,410 ± 174

Data are expressed as means ± SD (n = 3). Parent PAHs are in bold.

### 3.3. Mechanism of UVA Light-Induced Lipid Peroxidation by Nitro-PAHs

To determine the involvement of singlet oxygen in the peroxidation of methyl linoleate initiated by UVA irradiation of nitro-PAH, 3-nitro-BaP was selected for photoirradiation in the presence of a free radical scavenger or enhancer. For comparison, the parent PAH, BaP was tested in parallel. Sodium azide (NaN_3_) is a free radical scavenger and can effectively react with singlet oxygen (^1^O_2_) and hydroxyl radical [34,35]. Thus, for mechanistic studies, the use of NaN_3_ alone cannot determine whether singlet oxygen is involved in lipid peroxidation. Since singlet oxygen has a longer half-life in deuterated methanol (CH_3_OD) than in methanol [30,34], study of the effect by NaN_3_ and CH_3_OD provides a reliable approach for determining whether singlet oxygen is involved in peroxidation [34].

**Figure 5:**
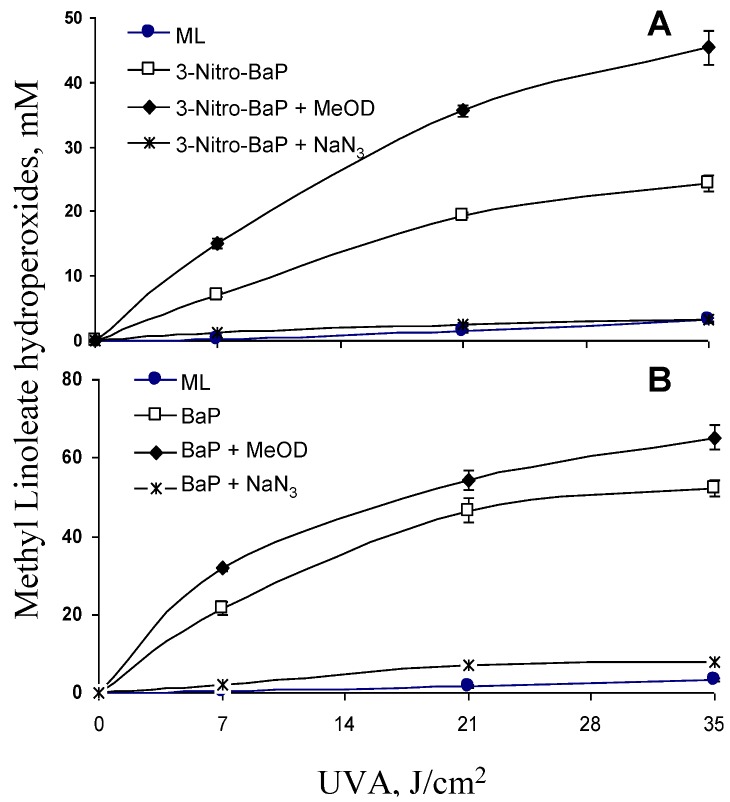
Effects of NaN_3_ and CH_3_OD on peroxidation of methyl linoleate (ML) initiated by (**A**) 3-nitro-BaP, and (**B**) BaP in CH_3_OH under UVA irradiation.

Lipid peroxidation induced by photoirradiation of 3-nitro-BaP with UVA at a dose of 35 J/cm^2 ^was inhibited 88% by NaN_3_ (*p* < 0.05) (Figure 5(A)). Lipid peroxidation increased 87% when CH_3_OH was replaced by CH_3_OD (*p* < 0.05) (Figure 5(A)). These results suggest that peroxidation of methyl linoleate initiated by photoirradiation of 3-nitro-BaP is mediated in part, if not all, by singlet oxygen. Similar results were obtained from BaP, which indicate that singlet oxygen is also involved in lipid peroxidation induced from UVA irradiation (Figure 5(B)).

### 3.4. Detection of Reactive Oxygen Species (ROS) and Free Radicals Using ESR

**Figure 6:**
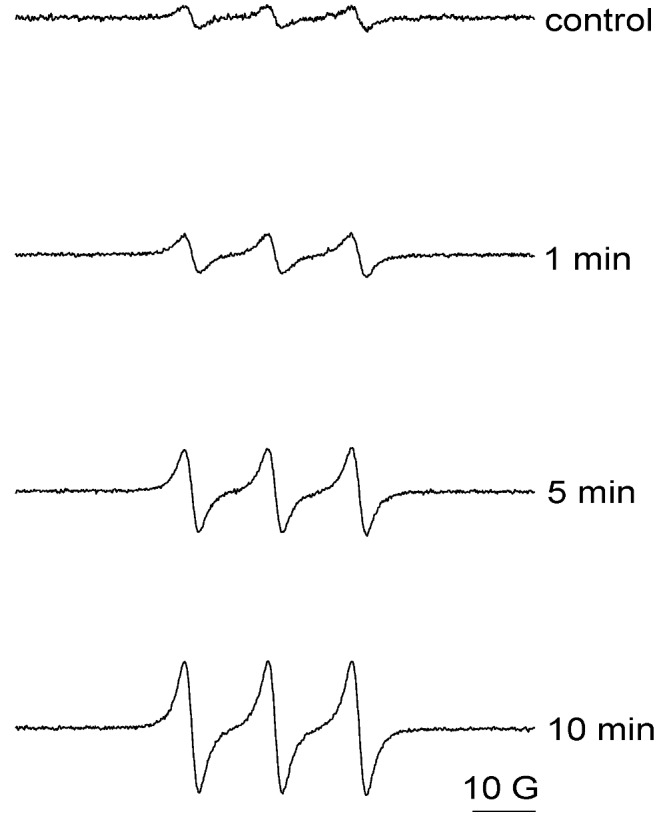
Generation of singlet oxygen from 4-nitropyrene. The sample containing 0.9 mM 4-nitropyrene and 20 mM 2,2,6,6-tetramethylpiperidine in 95% CH_3_CN was irradiated at 420 nm, and ESR signal recorded after 1, 5, and 10 min exposure. Control did not contain any 4-nitropyrene. ESR instrument settings were as follows: 20 mW microwave power, 100 G sweep width, 1 G field modulation amplitude, and 100 kHz modulation frequency.

ERS spin trapping was used to study the formation of ROS and free radicals by UVA irradiation of nitro-PAHs. The commonly used spin trap 2,2,6,6-tetramethylpiperidine (TEMP) was used to confirm singlet oxygen formation. It is well established that upon reaction of singlet oxygen with TEMP, the resulting 2,2,6,6-tetramethylpiperidine-1-oxyl (TEMPO) is a stable nitroxide, with a sufficiently long half-life to be detected by ESR spectroscopy [36,37]. In the first study, the sample containing 0.9 mM 4-nitropyrene (Figure 6) and 20 mM TEMP in 95% CH_3_CN was irradiated at 420 nm, and ESR signal recorded after 1, 5, and 10 min exposure. UVA light irradiation of TEMP alone did not result in an ESR signal (Figure 6 control). With concomitant exposure of both TEMP and 4-nitropyrene to UVA light for 1 min, singlet oxygen was generated, as evidenced by an ESR spectral profile that is typical of TEMPO (Figure 6) [31,34,35]. The intensity of ESR signals progressively enhanced when photoirradiation time increased to 5 and 10 min, respectively (Figure 6). These results suggest that photoirradiation of 4-nitropyrene with UVA light generates singlet oxygen in a light dose dependent manner. Similar results were obtained from the study of 6-BeP (data not shown).

UVA irradiation of 4-nitropyrene and 4-nitro-BeP in the presence of methyl linoleate was also conducted with NaN_3_ or CH_3_OD. The results suggest that singlet oxygen is generated from UVA irradiation (data not shown).

**Figure 7:**
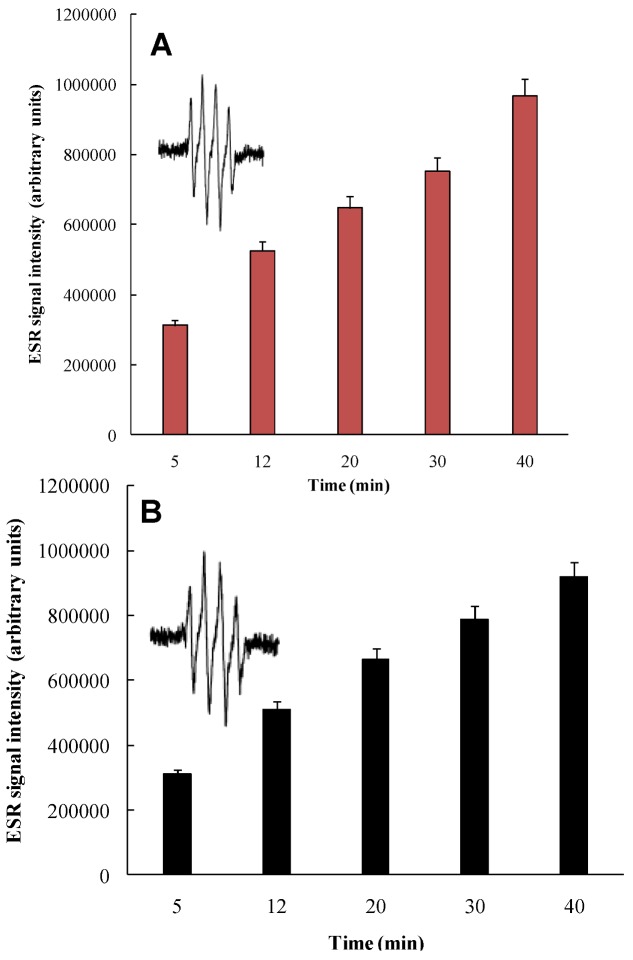
The generation of superoxide from (**A**) 4-nitropyrene and (**B**) 4-nitro-BeP. Samples contained 0.9 mM 4-nitropyrene or 4-nitro-BeP and 25 mM 5-*tert*-butoxycarbonyl-5-methyl-1-pyrroline-*N*-oxide (BMPO) in 90% CH_3_CN. Samples were irradiated at 420 nm, and ESR signal recorded after different time intervals. ESR instrument settings were as follows: 20 mW microwave power, 100 G sweep width, 1 G field modulation amplitude, and 100 kHz modulation frequency. The insert is the continuous wave ESR spectrum of BMPO-OH.

To determine whether UVA irradiation of nitro-PAHs generates superoxide radical anion, 5-*tert*-butoxycarbonyl-5-methyl-1-pyrroline-*N*-oxide (BMPO), a trapping agent that efficiently traps superoxide radical anion [31], was concomitantly irradiated with 4-nitropyrene or 4-nitro-BeP. UVA photoirradiation of BMPO with UVA light alone did not result in an ESR signal (data not shown). In addition, no ESR signal was observed when a solution of 4-nitropyrene or 4-nitro-BeP was mixed with BMPO in the absence of UVA light (data not shown). However, an ESR signal was observed after photoirradiation of 4-nitropyrene and BMPO with UVA light for 5 min (Figure 7A insert). The ESR spectral profile is identical to that produced from the reaction of xanthine with xanthine oxidase in the presence of BMPO [31,35], indicating that BMPO-OH adducts were formed and detected by ESR spin trapping methods. The intensity of these ESR signals progressively enhanced when photoirradiation time increased to 12, 20, 30, and 40 min, respectively (Figure 7). When superoxide dismutase (SOD), a superoxide radical scavenger, was added to a mixture containing 4-nitropyrene and BMPO, photoirradiation with UVA light for a period of 8 min produced no ESR signal (data not shown). These overall results suggest that UVA irradiation of 4-nitropyrene generates superoxide anion and that its quantity formed is dependent on the light dose.

Similar results were also obtained from UVA irradiation of 4-nitro-BeP under similar conditions (Figure 7(B)), which suggest that superoxide radical anion was generated from photoirradiation of 4-nitro-BeP with UVA light.

**Figure 8:**
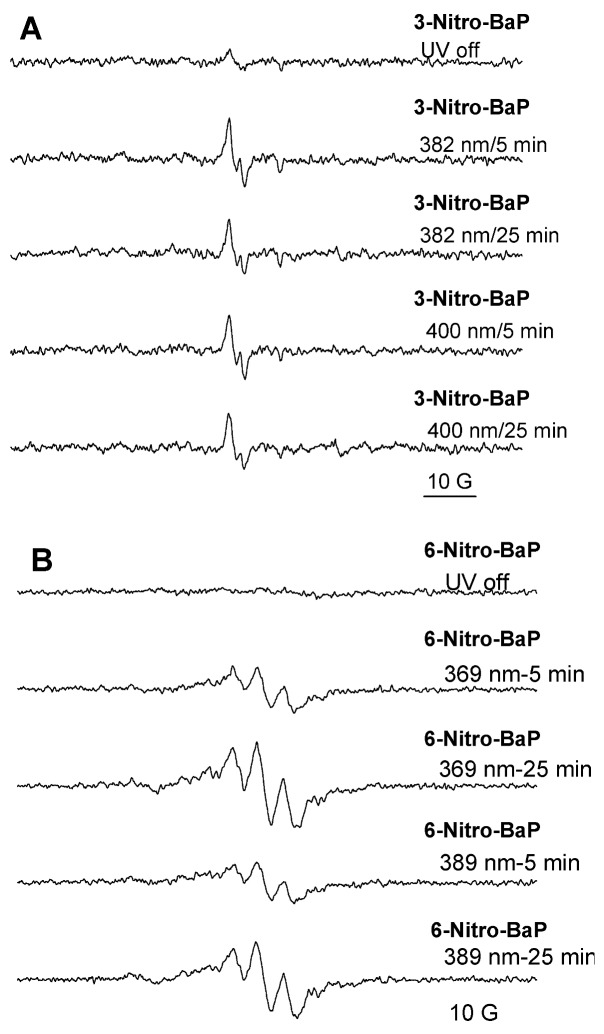
The generation of substrate-derived free radicals from (**A**) 3-nitro-BaP and (**B**) 6-nitro-BaP. Samples contained 0.9 mM 3-nitro-BaP or 6-nitro-BaP and 25 mM 5-*tert*-butoxycarbonyl-5-methyl-1-pyrroline-*N*-oxide (BMPO) in 90% CH_3_CN. Samples were irradiated at 382 and 400 nm for 3-nitro-BaP; and 369 and 389 nm for 6-nitro-BaP. ESR signal recorded after 5 and 25 min time intervals. ESR instrument settings were as follows: 20 mW microwave power, 100 G sweep width, 1 G field modulation amplitude, and 100 kHz modulation frequency.

ESR spectra were also collected to characterize radicals formed during UVA irradiation of 3-nitro-BaP and 6-nitro-BaP in the presence of 5,5-dimethyloxide pyrroline (DMPO). Upon reaction of DMPO with superoxide radical anion, the resulting DMPO-OOH adducts are unstable and easily decomposed to the corresponding hydroxyl DMPO-OH adducts [38]. As such, DMPO can be used to determine whether superoxide radical anion or hydroxyl radical generated. However, based on the ESR profiles, UVA irradiation of 3-nitro-BaP or 6-nitro-BaP in the presence of DMPO did not generate ESR signals of superoxide radical anion or hydroxyl radical (Figure 8). These results indicate that superoxide radical anion was not generated from photoirradiation of 3-nitro-BaP or 6-nitro-BaP with UVA light; while carbon-centered derived free radicals and/or PAH-oxy radicals were generated.

### 3.5. Computational Calculation of HOMO and LUMO Gaps

HOMO/LUMO gap is an important descriptor for the excited state property of organic molecules and usually directly affects their excited state reactions. Theoretical calculations of HOMO/LUMO energy gap of geometric isomers of nitro-BaPs, nitro-BePs and nitro-pyrenes were performed and compared with their corresponding photoinduced lipid peroxidation values. The calculation results are listed in Table 2. The results indicate that there is no correlation between the HOMO/LUMO energy gap and the level of the corresponding photoinduced lipid peroxidation.

**Table 2:** Quantum computation results of HOMO/LUMO energy gap of nitro-PAHs.

Compounds		HOMO (eV)	LUMO (eV)	HOMO/LUMO Energy gap (eV)	ML hydroperoxides (mM) at 7 J/cm^2^
**BaP series**					
	1-Nitro-BaP	−5.6129	−2.8589	2.754	753 ± 88
	2-Nitro-BaP	−5.5044	−2.6452	2.8592	346 ± 8
	3-Nitro-BaP	−5.6004	−2.8469	2.7535	1,205 ± 198
	6-Nitro-BaP	−5.6252	−2.6553	2.9699	1,003 ± 143
**BeP series**					
	1-Nitro-BeP	−5.8815	−2.6104	3.2711	171 ±15
	3-Nitro-BeP	−5.9286	−2.7867	3.1419	169 ± 6
	4-Nitro-BeP	−5.9106	−2.7579	3.1527	724 ± 13
**Pyrene series**					
	1-Nitropyrene	−5.8957	−2.8085	3.0872	1,612 ± 158
	2-Nitropyrene	−5.7732	−2.6093	3.1339	492 ± 16
	4-Nitropyrene	−5.8189	−2.7677	3.0512	610 ± 19

## 4. Discussion

In the paper, nitro-PAH-induced phototoxicity leading to lipid peroxidation was studied on the basis of structure-activity relationships. We first synthesized the isomeric 1-nitro-BaP, 3-nitro-BaP, 6-nitro-BaP, 1-nitro-BeP, and 3-nitro-BeP by novel approaches that are simple and convenient, providing all the compounds without contamination by their geometric isomers (Scheme 1 and Scheme 2). Subsequently, UVA photoirradiation of 19 environmental nitro-PAHs and their 10 parent PAHs in the presence of a lipid (methyl linoleate) was studied. The selected nitro-PAHs ranged from three to five aromatic rings. Seventeen of the nitro-PAHs exposed to UVA light induce lipid peroxidation in a light dose response manner (Table 1). In this study, the mechanisms of inducing lipid peroxidation were determined by using free radical scavengers or enhancers and by ESR spin trapping methodology. We found that the formation of lipid peroxidation was mediated by reactive intermediates including ROS (singlet oxygen and superoxide) and free radicals generated during UVA irradiation. It is noteworthy that although superoxide anion radicals were detected by the ESR-spin trapping approach, it was not detected by the enzyme inhibition reaction. Therefore, if superoxide is formed, it should be a very minor pathway.

For comparison, ten parent PAHs were also studied in parallel; a comparison indicated that the presence of a nitro group in the PAH molecule mostly decreases the level of lipid peroxidation, only 9-nitrophenanthrene caused more lipid peroxidation than phenanthrene. Three other nitro-PAHs, 1-nitropyrene, 6-nitrochrysene, and 2-nitrofluoranthene caused about the same amount of lipid peroxidation as their parent PAHs. We also determined that the level of lipid peroxidation formation was independent of the geometrical location and orientation of the nitro group, or the size of the PAH moiety in geenral. This is drastically different from the structure-activity study of mutagenicity and tumorigenicity of nitro-PAHs from which good correlations were observed [12,13,14,15,16,17,18]. For example, when assayed in *Salmonella typhimurium* tester strain TA98 in the absence of an exogenous S9 activation enzymes, nitro‑PAHs with the nitro substituent situated at the longest molecular axis, such as 2-nitro-BaP, exhibited the highest mutagenicity among the geometrical nitro-PAH isomers [17]. However, the induction of photo-induced lipid peroxidation by 2-nitro-BaP was lower than 1-, 3-, and 6-nitro-BaP (Figure 4). The relative levels of UVA-induced lipid peroxidation by the nitro-PAHs (Table 2) did not correlate with the relative carcinogenic activity or the tumor-initiation activity reported in the literature [11]. The lack of correlation is understandable since the mechanism leading to lipid peroxidation and the mechanisms involving enzymatic metabolic activation leading to tumorigenicity are different.

The LUMO/HOMO energy gap of the isomeric nitro-BaPs, nitro-BePs, and nitropyrenes was calculated, and there was no correlation with the level of induced lipid peroxidation (Table 2), although we previously found that HOMO/LUMO can be used to correlate with UVA-induced DNA cleavage by the twelve isomeric methylbenz[*a*]anthracenes [39]. The lack of correlation may be due to the fact that induction of lipid peroxidation is mediated by ROS, rather than through only simple photo-excitation from ground to excited state. Lipid peroxidation is probably directly related to singlet oxygen and superoxide quantum yield and life time as a result of the irradiation and the solvent environment, but these quantum yields and life times may not directly relate to the HOMO-LUMO gaps. Once a nitro-PAH is in the excited singlet state, its intersystem crossing rate *versus* the fluorescence and other non-radiative decay rates will determine the triplet state concentration, which, due to its longer lifetime, is the source for singlet oxygen and superoxide. Also, the excited state nitro-PAH, singlet or triplet, may undergo reactions with other molecules than oxygen. Therefore, our theoretical calculations on HOMO-LUMO gap provide the evidence that HOMO-LUMO gap is not a decisive factor in the formation of photo-induced lipid peroxidation by nitro-PAHs. Recently, there have been several reports on the photophysics of nitro-PAHs [40,41,42,43]. An ultrafast intercrossing was observed from the excited singlet state to the excited triplet state. It was concluded that the presence of the nitro-group greatly enhances the intersystem crossing rate in comparison to the parent PAHs. The triplet state nitro-PAHs can react in many ways: (i) lose energy to go back to the ground state, (ii) formation of phenoxy radicals and NO, which either recombine or form nitroso-substituted products or the phenoxy radical reacts with another molecule to extract a hydrogen to form phenolic compounds, (iii) react with ground state oxygen to form ROS. Both reactions (i) and (iii) may produce species leading to lipid peroxidation. We previously studied the photolysis of 9-methyl-10-nitroanthracene and 12-methyl-7-nitrobenz[*a*]anthracene [44]. Both nitro-PAHs have nitro functional groups with an orientation perpendicular to the aromatic moiety. Similar to the finding previously reported by Chapman *et al.* [45], we found that the excited state rearrangement reaction, which involves the rearrangement of the nitro group to a nitrite, followed by breaking of the N-O bond producing NO radical. The NO radical further forms a bond with the carbon on the opposite site of the benzene ring through radical recombination.

**Figure 9:**
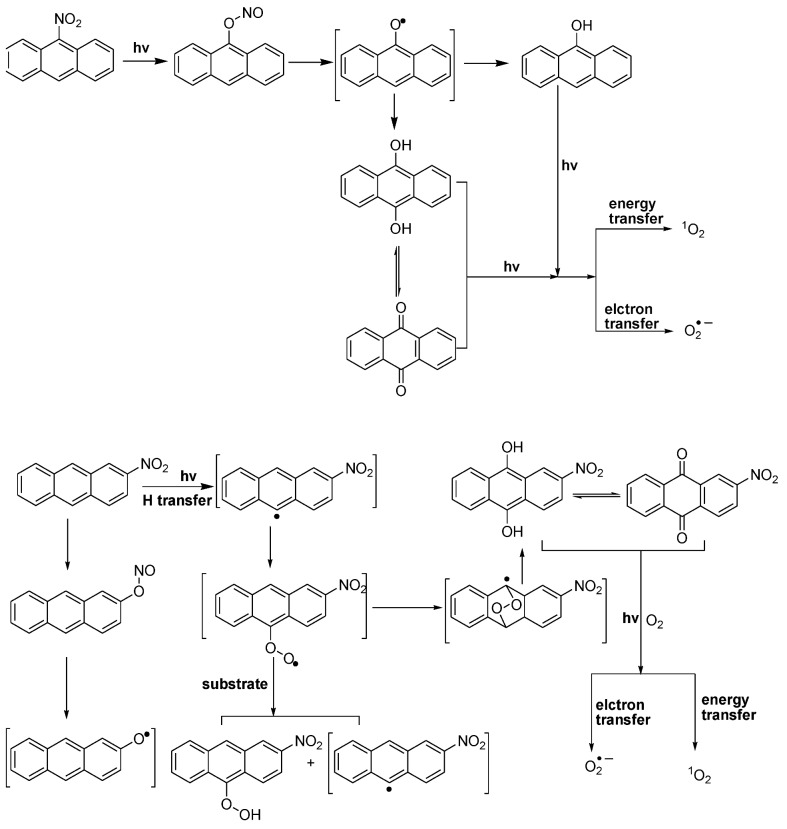
Using 2- and 9-nitroanthracenes as examples to show the proposed mechanism of inducing singlet oxygen, superoxide anion radicals, and carbon-centered free radicals by UVA irradiation of nitro-PAHs.

The complex photo-induced reactions of nitro-PAHs can be further illustrated by photoirradiation of 2- and 9-nitroanthracene as examples (Figure 9). 2-Nitroanthracene and 9-nitroanthracene are arbitrarily selected to represent nitro-PAHs with their nitro group adopting an orientation perpendicular or parallel to the aromatic moiety. As shown in Figure 9, the postulated free radicals formed at least include ROS (singlet oxygen and superoxide), PAH-oxy radicals, nitro-PAH-oxy radicals, and nitro-PAH carbon-centered radicals. This can be supported by our present study that not only singlet oxygen (Figure 5 and Figure 6) and superoxide (Figure 7) are generated, but possibly oxy radicals and/or carbon-centered radicals as well (Figure 8).

Nitro-PAHs are environmental contaminants and concomitantly exposed to sunlight. It is significant to find out whether or not nitro-PAHs are phototoxic. Since lipid peroxidation in humans has been associated with many diseases including cancer, athereosclerosis, ischemia, inflammation, liver injury, aging, *etc.*, it is significant to determine whether photoirradiation of nitro-PAHs with UVA light (sunlight) can initiate lipid peroxidation, and the cause of its formation. It has long been known that lipid peroxidation can lead to induction of tumors in experimental animals [46,47]. ROS can also damage DNA and proteins leading to aging, inflammation, cardiovascular diseases, cancer and other age-related diseases [48,49,50,51]. On the basis of our present results, people exposed to the nitro-PAHs containing environmental pollutants on the skin with concomitant exposure to sunlight may result in deleterious effects if the level of the nitro-PAHs is high and if sunlight exposure is long. To assess human health risks posed by environmental phototoxic nitro-PAHs, more investigations on animals and ultimately on humans are warranted.
